# Salivary extracellular vesicle-derived microRNAs are related to variances in parameters of obesity, taste and eating behaviour

**DOI:** 10.1016/j.molmet.2025.102265

**Published:** 2025-10-03

**Authors:** Kristin Röhrborn, Anne Hoffmann, Andrea Lorenz, Peter Kovacs, Tobias Hagemann, Paul Czechowski, Maria Sehm, Annette Horstmann, Michael Stumvoll, Matthias Blüher, Imke Schamarek, Kerstin Rohde-Zimmermann

**Affiliations:** 1 Helmholtz Institute for Metabolic, Obesity and Vascular Research (HI-MAG), Helmholtz Center Munich at the University Leipzig and the University Clinic Leipzig, AöR, 04103 Leipzig, Germany; 2 Department of Prosthodontics and Materials Science, University of Leipzig, 04103 Leipzig, Germany; 3 Department of Medicine III, Division of Endocrinology, Nephrology and Rheumatology, University of Leipzig, 04103 Leipzig, Germany; 4 Department of Neurology, Max Planck Institute for Human Cognitive and Brain Sciences, 04103, Leipzig, Germany; 5 Department of Psychosomatic Medicine and Psychotherapy, Leipzig University Medical Center, 04103 Leipzig, Germany; 6 Department of Psychology, Faculty of Medicine, University of Helsinki, 00014 Helsinki, Finland

**Keywords:** Saliva, Extracellular vesicles, microRNA, Taste, Obesity, Eating behaviour

## Abstract

**Background:**

Extracellular vesicles (EVs), conveyors of microRNAs, have recently been linked to obesity. As taste is a potent driver of eating behaviour and food intake, it's connection to EVs is of increasing interest. This study aimed at deciphering the salivary EV-microRNA profile in relation to taste perception and metabolic pathways of obesity.

**Methods:**

Small RNA sequencing was performed on isolated salivary EVs of 90 participants from the Obese-Taste-Bud study. Pathway enrichment and association analyses were conducted to link identified microRNAs to taste recognition, eating behaviour, food intake and various anthropometric-, metabolic- and oral health parameter.

**Results:**

The 626 identified microRNAs clustered into pathways related to energy regulation, obesity and diabetes, cell signaling and taste perception. The top three enriched microRNAs are miR-1246, miR-1290 and miR-148a-3p which showed significant associations with fasting blood glucose and cholesterol level, anthropometrics and blood pressure (p < 0.05). Additionally, these microRNAs associate with trait eating behaviour (p < 0.05). Several other microRNAs were linked to differences in taste recognition scores and are further related to parameters of glucose metabolism and periodontal health, salivary insulin level or food intake (p < 0.05).

**Conclusions:**

This study, one of the largest on salivary EVs, supports an interrelation of EV's microRNA load with metabolism, eating behaviour and taste recognition offering potential targets for obesity intervention.

## Highlights

• Salivary EV-derived microRNAs are enriched in pathways related to obesity including taste recognition and food intake.
• Levels of microRNAs are related to metabolic parameters, taste recognition scores, trait eating behaviour and food intake.
• Most enriched microRNAs (miR-1246, -1290 and -148a-3p) associate with anthropometrics, blood glucose and trait restraint.

## 1 Introduction

An emerging area of research focuses on extracellular vesicles (EVs), which are tiny membrane-bound particles secreted by all cell types of the body and carrying important biomarkers and signaling molecules. Particularly adipose tissue derived circulating EVs have gained attention for their role in driving inflammatory processes upon obesity [[1], [2], [3]]. Beyond that, they affect eating behaviour through their ability to cross the blood–brain barrier [4]. It has been demonstrated that visceral adipose tissue (VAT) derived EVs from obese mice increase food consumption with consequent weight gain when being administered to mice consuming a standard chow diet. The researchers linked this to a transfer of microRNAs and long non-coding RNA from VAT-EVs to hypothalamic neurons. With this, they act on critical signaling pathways relevant to activate food intake and are thereby mediators of the adipocyte-neuron crosstalk [1].

In general, EVs play a crucial role in modulating local immune responses, facilitating communication between cells, and maintaining overall health by circulating throughout the body [5,6]. Recently, we showed that particularly the level of salivary EVs is related to obesity [7]. Salivary EVs may partially derive from blood, but do more likely originate from local sources, specifically the salivary glands and tongue tissues [[8], [9], [10]]. An altered salivary environment in obesity including changes in the EV composition can promote inflammation, creating a cycle that negatively impacts oral health, taste perception, eating habits and ultimately metabolic and overall health status [[10], [11], [12], [13]]. The EV-derived miR-25-3p for instance is implicated in the progression of periodontitis and was shown to be enriched in patients suffering from obesity and type II diabetes [14]. In the pharyngeal region of the developing mouse, the miR-200-deltaNotch and fibroblast growth factor (FGF) signaling axis, factors for cell differentiation, play a crucial role in controlling taste bud formation pointing towards the regulatory role of microRNAs in taste function [15]. The delivery of microRNAs through salivary EVs could thereby also regulate adult taste tissue and contribute to the observed challenges in taste sensation up to altered eating behaviour during obesity [16,17]. Moreover, obesity associated inflammation, especially a rise in tumor necrosis factor alpha (TNFa) level, contribute to a reduction of taste cell proliferation, hence regeneration [11,18]. This systemic low grad inflammation may also impair the function of surrounding non-taste epithelium or cranial nerves innervating both, taste and non-taste cells which consequently would affect structure, mechanosensory function or taste transmission [19,20]. In addition, an altered gene expression level in taste buds were found between lean and obese subjects where salivary EVs might explain some of these aspects [21].

Understanding the interconnection of obesity, oral health, salivary EVs and taste perception is of potential relevance for the prevention and management of obesity-related complications likely by influencing eating behaviour and food consumption. Therefore, the aim of the present study was to address the microRNA profile of salivary EVs in a cohort of participants with normal-, overweight and obesity and relate this to taste recognition, clinical biomarkers of obesity and metabolic health and factors relevant for oral health and eating behaviour. Future research in this field may provide insights into how salivary EVs could serve as biomarkers for early detection or as therapeutic targets to improve oral health and potentially mitigate some of the effects of obesity.

## 2 Material and methods

### 2.1 Study cohort

A total of 90 participants from the Obese Taste Bud (OTB) Study (NCT04633109; DRKS00022950) were included for the present analyses and main characteristics are given in Table 1 [22]. The Study was conducted at the University Hospital Leipzig in collaboration with the Helmholtz Institute for Metabolic, Obesity and Vascular Research (HI-MAG) as a cross-sectional observational study, performed in accordance with the Declaration of Helsinki and approved by the Ethics Committee of the University of Leipzig. People with a body mass index (BMI) over 18 and aged between 18 and 69 years were recruited after they gave written informed consent and who did not suffer from taste- and smell disorders or any severe diseases, as described elsewhere [22]. The participants were extensively characterized which included the performance of a standardized taste test using commercially available taste strips (Burckhart ODOFIN taste strips, Medisense, Stuttgart, Germany). The test consisted of four increasing concentrations per sweet, sour, salty, bitter and umami tastes and allowed the definition of taste recognition scores per single taste quality. A sum-score for the total taste recognition ability was calculated including all taste qualities, except umami as recommended by the manufacturer. Furthermore, data obtained included the assessment of lingual fungiform taste bud density in a defined area of 177 mm^2^ of the anterior tongue, salivary pH level using indicator paper, and the periodontal health status by using the periodontal screening and recording (PSR) score according to clinical standards. Data on body fat using bioimpedance analyses were obtained next to anthropometric data (body height and weight, BMI, waist and hip circumferences, waist-to-hip ratio (WHR) and blood pressure (BP) measurements. Further, self-report questionnaires were used to assess data for trait eating behaviour (German version of the three-factor eating questionnaire [23,24]) and food consumption. Following previously established procedures, the eating behaviour traits “cognitive restraint”, “disinhibition” and “hunger” were formed with the additional subscales “uncontrolled eating”, “emotional eating” and “restraint eating” [[24], [25], [26]]. All items are additionally coded as “yes” or “no”. A food frequency questionnaire was used through which the intake of total kilocalories (kcal), carbohydrates, fat, protein and salt was obtained. Biomaterial included a fasting blood draw and saliva sampling after >12 h overnight fasting. Serum and plasma samples were either kept on ice before further preparation and analysis, or were analyzed at the Institute of Laboratory Medicine, Clinical Chemistry and Molecular Diagnostics (ILM) of the University of Leipzig Medical Center. Investigated parameters included serum glucose, insulin (and calculated Homeostasis Model Assessment-Insulin Resistance; HOMA-IR), glycated hemoglobin (HbA1c), and lipid levels among others as stated elsewhere [22]. Enzyme-linked Immunosorbent Assays (ELISA) were performed for the assessment of inflammatory markers, leptin, adiponectin and insulin in serum and/or saliva [7,22]. A detailed description on data assessment and the study protocol is provided elsewhere [22].

**Table 1:** Characteristics of the study cohort.

Clinical/metabolic parameter	N	total cohort	N	normal weight	N	obese	P
Body height (m)	90	1.71 (1.63/1.77)	38	1.75 (1.63/1.77)	34	1.7 (1.62/1.75)	0.304
Body weight (kg)	90	82 (69.5/102.1)	38	62.2 (57.6/77.6)	34	110.8 (91.12/134.95)	<0.001
BMI (kg/m^2^)	90	27.26 (22.76/33.64)	38	22.3 (20.16/23.67)	34	37.1 (31.02/46.32)	<0.001
Waist (cm)	90	91 (73/103.5)	38	73 (70/80)	34	104.5 (93.75/124.25)	<0.001
Hip (cm)	90	102 (92/121)	38	91 (87/98)	34	122.25 (106.88/137)	<0.001
WHR	90	0.85 (0.77/0.93)	38	0.81 (0.75/0.87)	34	0.86 (0.82/0.95)	<0.001
Fat mass (%)	76	30.03 (21.88/42.61)	36	21.9 (18.77/29.35)	26	45.58 (41.05/55.30)	<0.001
Systolic BP (mmHg)	50	117 (107/132)	16	112 (106/122)	21	131 (114/135.5)	0.012
Diastolic BP (mmHg)	50	78 (72/85)	16	75 (70/81)	21	83.5 (76.5/91.5)	0.023
FPG (mmol/mol)	88	4.69 (4.46/4.98)	37	4.49 (4.37/4.79)	33	4.845 (4.64/5.23)	<0.001
FPI (pmol/l)	75	40 (28.1/83.7)	32	28.1 (22.9/35.6)	28	81.1 (58.12/122.25)	<0.001
HbA1c (%)	86	5.3 (5.1/5.6)	35	5.2 (5.1/5.4)	33	5.35 (5.1/5.75)	0.051
Total Chol (mmol/l)	76	4.65 (4.05/5.64)	32	4.61 (3.77/5.42)	28	4.68 (4.27/5.42)	0.441
HDL-Chol (mmol/l)	76	1.47 (1.2/1.96)	32	1.93 (1.5/2.16)	28	1.38 (1.15/1.51)	<0.001
LDL-Chol (mmol/l)	76	2.88 (2.29/3.71)	32	2.32 (1.9/3.61)	28	2.93 (2.44/3.61)	0.064
TG (mmol/l)	76	0.99 (0.75/1.41)	32	0.78 (0.67/0.87)	28	1.24 (0.97/1.78)	<0.001
FFA (mmol/l)	76	0.62 (0.45/0.81)	32	0.58 (0.45/0.79)	28	0.8 (0.63/0.84)	0.328

Data are presented as median (25th/75th percentile). P < 0.05 were considered statistically significant and were calculated using Mann-Whitney-test. N = number, BMI = body mass index, WHR = waist to hip ratio, FPG = fasting plasma glucose, FPI = fasting plasma insulin, Chol = Cholesterol, HDL = high density lipoprotein, LDL = low density lipoprotein, TG = Triglycerides, FFA = free fatty acids, BP = blood pressure.

### 2.2 Saliva sampling and EV preparation

Sample collection, the preparation and the characterization of salivary EVs have been described in detail elsewhere [7]. Briefly, participants were instructed to stay fasted and refrain from drinking, smoking, chewing gum or brushing their teeth for at least 30min before approximately 2 ml actively secreted saliva was collected by spitting into sterile falcon tubes. The collected samples were kept on ice and processed within 1.5 h after sampling by centrifugation at 3500*g* for 20 min at 4 °C to remove debris and were then stored at −80 °C. The EVs were subsequently isolated from the remaining supernatant via size exclusion chromatography (SEC) using the ÄKTA Purifier 10 system (GE Healthcare, Munich, Germany) with a Cytiva Superdex™ 200 Increase 10/300 GL column and phosphate-buffered saline (PBS) as the elution buffer. The fractions between 10 and 600 kDa were pooled per participant, which were considered the fractions containing EVs <1000 nm in size based on Nanosight analyses of isolated EVs conducted earlier [7]. Samples were concentrated with Vivaspin® 6 columns (Sartorius, Göttingen, Germany) and stored at −80 °C before further analyses.

### 2.3 RNA isolation and small RNA sequencing of salivary EVs

Total RNA was extracted from 200 μl of the isolated salivary EV fraction using the miRNeasy Micro Kit (Qiagen, Hilden, Germany) by following the instructions provided by the manufacturer. A total of 6 μl of isolated EV-derived RNA was processed for small RNA library preparation using the NEB Small RNA Library Prep kit from Illumina® (New England Biolabs, Ipswich, USA). Libraries were sequenced in SE150 mode on a NovaSeq6000 platform (Illumina, San Diego, USA).

### 2.4 Data processing and RNA annotation

Small RNA raw sequencing data were processed using the Nextflow v24.10.1 pipeline nf-core/smrnaseq v2.4.0 [27]. Briefly, adapter sequences were trimmed, and quality control was performed on the reads before aligning them against the human (GRCh38) miRBase (https://mirbase.org/) mature microRNA sequences using Bowtie v1.3.1 [28]. Contaminant-filtered and collapsed reads were subsequently aligned against the miRBase hairpin sequences. Based on the alignments, known microRNAs were annotated using miRTOP v0.4.28 (http://mirtop.github.io). Additionally, MiRDeep2 v0.1.3 was employed, which includes mapping against the reference genome with its mapper module [29]. The results were filtered based on significant p-values derived from Randfold, which evaluates the likelihood of folding into hairpin structures, and required a true positive probability of ≥50% [30]. For each microRNA, the highest count value among its precursors was utilized. In total, 626 known microRNAs were identified: 82 were annotated by both miRTOP and MiRDeep2, while 28 microRNAs were exclusively detected by MiRDeep2 and 516 by miRTOP alone. Only microRNAs with counts greater than 10 reads were considered for annotation.

To analyse the RNA composition of salivary EVs, reads that could not be assigned to microRNAs were mapped to the human reference genome using Bowtie, allowing for a maximum of two base pair mismatches. Successfully mapped reads were then aligned to the RNAcentral database v24.0 (http://rnacentral.org/) for annotation against a comprehensive collection of non-coding RNA sequences [31]. Reads that could not be mapped to the RNAcentral database were subsequently aligned to complementary DNA sequences to identify and classify reads originating from mRNA. Both alignment steps were performed with Bowtie, permitting one mismatch.

### 2.5 Pathway enrichment analyses

MicroRNAs adopted gene set enrichment analysis (GSEA) for the identified salivary EV-derived microRNAs was conducted using MiEAA 2.1 (https://ccb-compute2.cs.uni-saarland.de/mieaa/, accessed on December 12, 2024). The KEGG and mirWALK databases were selected for pathway predictions, with false discovery rate (FDR; Benjamini-Hochberg) adjustments applied [32,33]. All microRNAs with a read depth greater than 10 and expressed in at least one sample (N = 90) were included in the analyses to provide a comprehensive overview of the functions of the detected salivary EV-derived microRNAs. The enriched pathways were grouped into six categories by functional relatedness and to highlight the most relevant for the present analyses: “metabolism”, “energy regulation”, “obesity and diabetes mellitus”, “immune defense and inflammation”, “cellular signaling” and “taste signaling”.

### 2.6 Data filtering for association analyses

To investigate the relationship between salivary EV-derived microRNAs and anthropometric as well as metabolic parameters, we employed a series of filtering steps to ensure the robustness and reliability of our expression data. The microRNA expression data were normalized using the trimmed mean of M values (TMM) method implemented in edgeR v4.4.2 [34]. However, we observed significant variation in the detected microRNAs expression depth across samples. Aiming to provide reliable expression estimates, we mitigated potential biases in our analyses by removing samples that expressed fewer than 16 distinct microRNAs or that had fewer than 10,000 total reads across all microRNAs resulting in N = 63 samples. Despite this reduction, only a limited number of microRNAs were consistently expressed across multiple samples. Some microRNAs were exclusively present in samples with high expression levels, likely due to challenges associated with RNA extraction from salivary EVs and small RNA sequencing [35], as their abundance can vary with EV purity, RNA extraction efficiency, and library preparation [36]. To improve the signal-to-noise ratio, we utilized the filterByExpr function from the edgeR R package to improve data quality and by following similar application elsewhere [34,37]. This function filters out microRNAs that do not meet a minimum expression threshold while considering BMI groupings. As a result of this filtering process, 39 microRNAs remained for phenotype association analysis. It is important to note that without these stringent filtering steps, including the removal of low-expressed microRNAs, meaningful correlation analyses would not have been feasible. Although TMM normalization addresses read count discrepancies, it does not compensate for the lack of robust expression data from low-abundance microRNAs. Thus, our methodological approach ensures that only high-quality and relevant data are included in subsequent analyses.

### 2.7 Statistical analyses

Characteristics of the study participants are presented as median values with 25th and 75th percentile. To assess differences between participants with and without obesity, a Mann–Whitney U test was conducted using SPSS statistics software v29.0.2.0. Spearman's rank correlation coefficient was calculated to evaluate the relationship between continuous parameters and microRNA expression values, utilizing the RVAideMemoire R package v0.9–83.7. Additionally, an Analysis of Variance (ANOVA) was performed to examine the relationship between categorical variables and microRNA expression, employing the stats package in R v4.4.1. Analyses were carried out in R v4.4.1 (https://www.R-project.org/) [38]. P-values unadjusted for multiple testing (excepted GSEA) were considered significant at < 0.05.

## 3 Results

### 3.1 Salivary extracellular vesicle RNA composition

**Figure 1:**
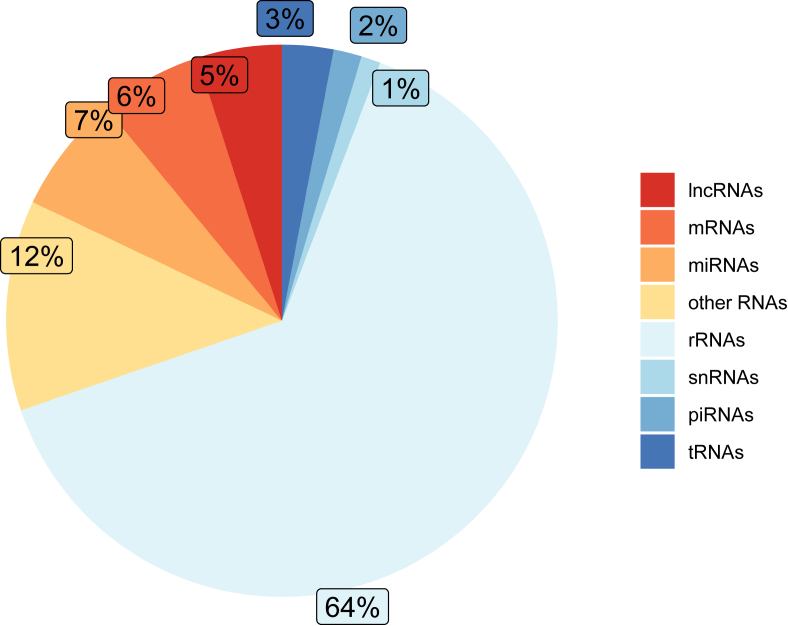
**Salivary extracellular vesicle RNA composition.** The pie-chart highlights the percentage distribution of various RNA types identified in the salivary extracellular vesicle (EV) cargo, based on small RNA-seq data derived from N = 90 participants. lncRNA = long non-coding RNA; mRNA = messenger RNA; miRNA = microRNA; rRNA = ribosomal RNA; snRNA = small nuclear RNA; piRNA = piwi-interacting RNA: tRNA = transfer RNA.

Salivary EVs were isolated from 90 participants and subjected to small RNA sequencing to identify the RNA landscape of these particles. Although all samples met the established sequencing quality criteria, an average of 75 % of the reads were either too short or could not be mapped to the human genome. This finding aligns with previous studies that have reported similar challenges in mapping and annotating small RNA sequences due to their inherent characteristics [35,39,40]. Furthermore, on average, 57 % of the mapped reads could not be classified into specific RNA types, suggesting that a significant portion of the salivary RNA may represent novel, uncharacterized species. Thus, an overview of the RNA cargo in salivary EVs by utilizing established annotation methods for diverse RNA types is illustrated in Figure 1. Regarding the genomically mapped and RNA-classifiable reads, the majority of the salivary EV cargo is composed of rRNA (64%), followed by microRNA (7 %), mRNA (6 %), and lncRNAs (5 %). Other small RNAs, including snoRNAs, yRNAs, and scRNAs, along with piRNAs, snRNAs, and tRNAs, collectively account for 18 % of the total RNA content. It has widely been shown that especially microRNAs packed into EVs can alter the function of their recipient cells. Therefore, we focused on the microRNA composition of salivary EVs to understand their potential contribution to oral and overall heath in obesity as well as taste recognition and trait eating behaviour. EV-derived microRNAs were identified using MiRDeep2 and miRTOP which resulted in the identification of a total of 626 different salivary EV-derived microRNAs (Supplementary Table 1).

### 3.2 Key roles of salivary EV-derived microRNAs in metabolic regulation, obesity, cell signaling and taste perception

**Figure 2:**
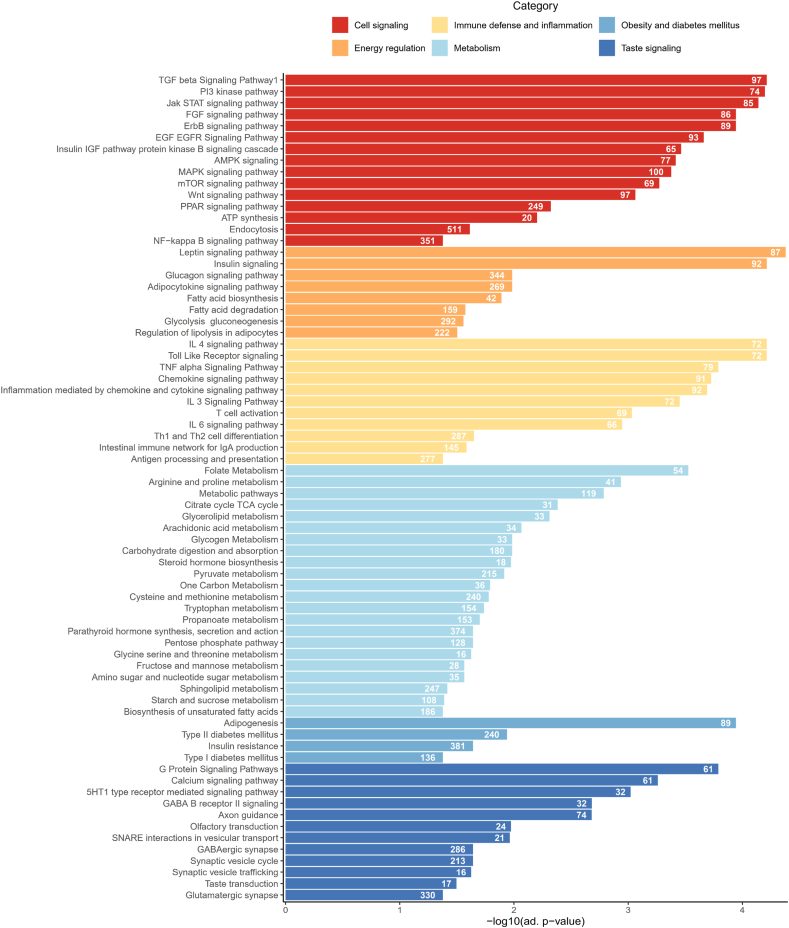
**Key findings on pathway enrichment for salivary EV-derived microRNAs.** Pathways being closely related to particular functions were grouped into six categories of interest: energy regulation, cell signaling, obesity and diabetes mellitus, taste signaling, immune defense and inflammation, and metabolism. The numbers in the bars correspond to the count of included salivary EV-derived microRNAs. Gene set enrichment analysis adapted for miRNAs was based on the KEGG and mirWALK databases, with adjustments made for false discovery rate (FDR). All significantly enriched pathways identified are listed in Supplementary Table 1. A total of 90 salivary EV-enriched microRNAs were included in the analysis.

To capture the potential functional trail of the identified 626 microRNAs in the 90 samples, KEGG and mirWALK pathway enrichment analyses were performed. The pathways being of highest relevance for the present analyses were grouped into six categories based on related physiological function. Of highest relevance are pathways related to overall metabolism, obesity, oral health and taste and are presented in Figure 2 while the whole list is given in Supplementary Table 1. Of all identified EV-microRNAs, 82 % (511 microRNAs) are related to endocytosis, a process through which cells internalize molecules including nutrients [41]. The majority of enriched pathways belong to metabolic processes and cell signaling. Interestingly, several pathways contributing to taste signaling were identified, as well as a high number of inflammatory and obesity/energy regulating pathways. Overall, the identified pathways highlight a role of salivary EV-derived microRNAs in various metabolic and signaling processes being known to contribute to the obesogenic phenotype.

### 3.3 Salivary EV-derived microRNA dynamics on metabolic traits

**Figure 3:**
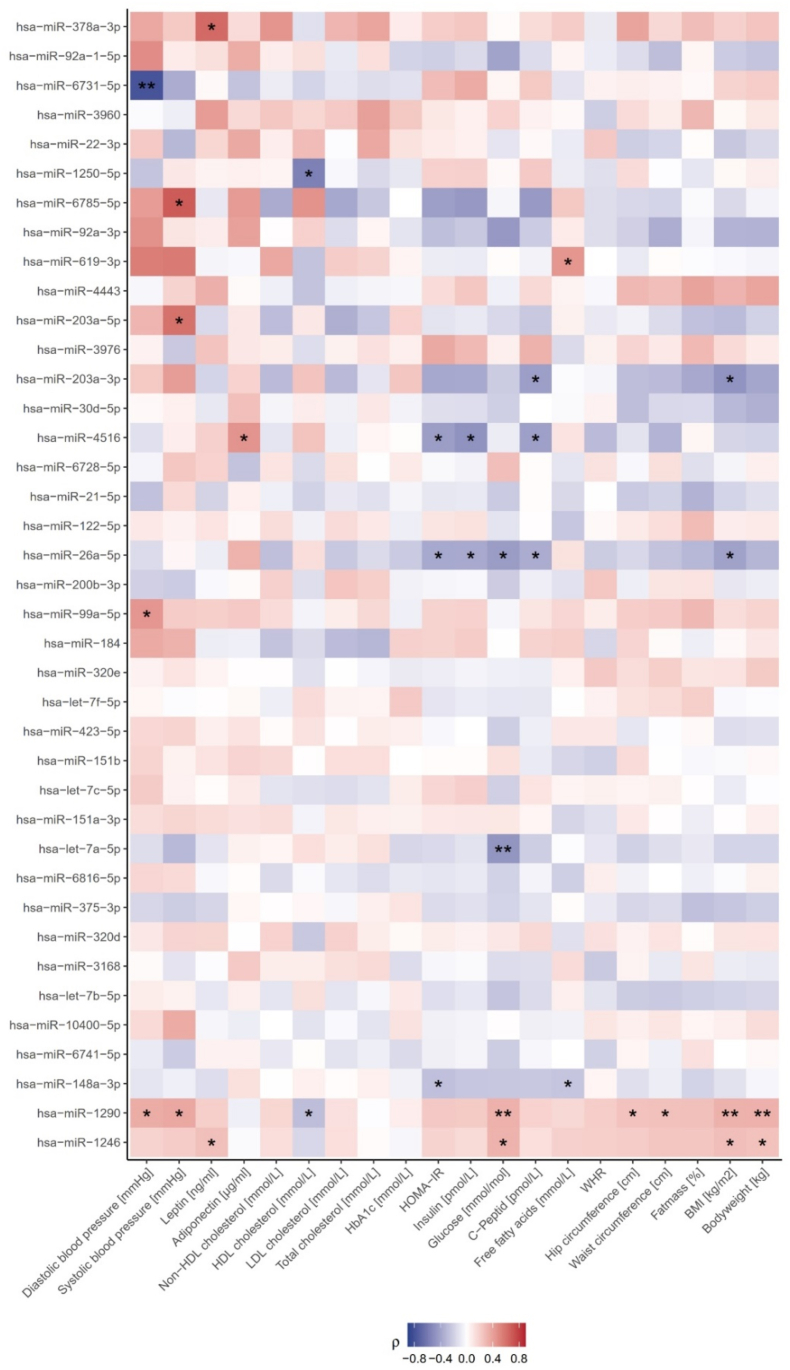
**Salivary EV-derived microRNA dynamics on metabolic traits.** Spearman correlation analyses were performed for 39 expressed salivary EV-derived microRNAs. Strength of the correlation coefficients is color-coded, ranging from negative (blue) to positive (red). Significance levels are indicated as ∗ <0.05 and ∗∗ <0.01. HDL = high density lipoprotein; LDL = low density lipoprotein; HbA1c = glycated hemoglobin; HOMA-IR = Homeostasis Model Assessment-Insulin Resistance; WHR = waist to hip ratio; BMI = body mass index.

Furthermore, we aimed to investigate whether salivary EV-derived microRNAs are related to metabolic, taste and eating related parameters. To maintain data integrity essential for reliable correlation analyses, we applied rigorous quality filtering criteria, which led to the exclusion of 27 samples due to insufficient microRNA expression or lower read counts. Such stringent filtering is particularly important in EV-derived datasets, where RNA abundance is more strongly influenced by EV purity, extraction efficiency, and library preparation compared to most other biofluids [36]. Subsequently, the analyses were restricted to 39 microRNAs that were consistently highly expressed across all remaining samples, thereby ensuring precise and meaningful correlation assessments within a dataset of 63 participants. Among those, the most enriched microRNAs include miR-1246, miR-1290, and miR-148a-3p. Notably, two specific salivary EV-derived microRNAs, namely miR-30 d-5p (F = 4.5, P = 0.0453) and miR-320e (F = 5.5, P = 0.0254), exhibit significant differences in expression between individuals with and without obesity, while gender differences were observed for miR-10400-5p (F = 4, P = 0.049) and miR-3168 (F = 4.1, P = 0.0479). In addition, several microRNAs are correlated with anthropometric and metabolic parameters: For instance, the highly expressed salivary EV-derived microRNA miR-1290 positively correlates with metabolic and obesity traits such as BMI (r = 0.3, P = 0.0093), body weight (r = 0.3, P = 0.0088), waist- (r = 0.3, P = 0.0371) and hip (r = 0.3, P = 0.0433) circumference, fasting plasma glucose levels (r = 0.3, P = 0.0089), or increased systolic (r = 0.4, P = 0.0358) and diastolic (r = 0.4, P = 0.0482) blood pressure. MiR-1246 positive correlates with BMI (r = 0.3, P = 0.0292), body weight (r = 0.3, P = 0.0423), fasting plasma glucose (r = 0.3, P = 0.0105), and leptin levels (r = 0.3, P = 0.0396). In contrast, miR-148a-3p exhibits opposing effects, being significantly negatively correlated with HOMA-IR (r = −0.3, P = 0.034) and free fatty acids levels (r = −0.3, P = 0.0488) in blood. Further, microRNAs being negatively related to BMI are miR-26a-5p (r = −0.4, P = 0.0225) and miR-203a-3p (r = −0.5, P = 0.0188) which are also linked to sugar metabolism. An overview of all microRNAs is provided (Figure 3; statistics in Supplementary Table 2), whereas Supplementary Fig. 1 shows only those miRNAs highlighted in the text.

### 3.4 Associations of salivary EV-derived microRNAs with oral health, systemic inflammation and taste recognition

**Figure 4:**
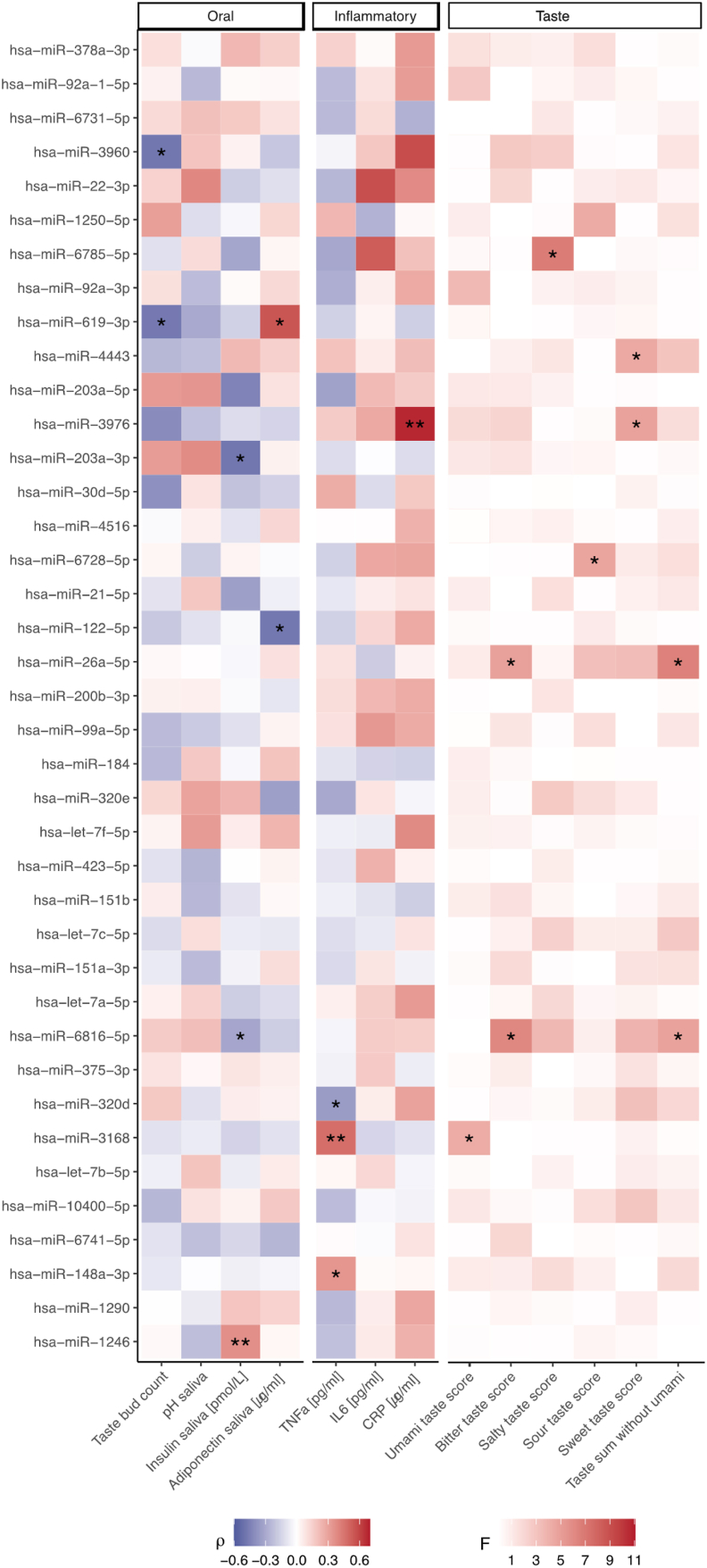
**Associations of salivary EV-derived microRNAs with oral health, systemic inflammation and taste recognition.** Spearman correlation analyses were performed to assess for associations between microRNA expression and “oral” (left panel) and “inflammatory” (middle panel) markers. Using ANOVA, group differences of the miRNAs expression across taste scores (right panel) were addressed. A total of 39 salivary EV-enriched microRNAs were included in the analysis. The strength of the correlation coefficients is color-coded, ranging from negative (blue) to positive (red), with shades of red indicating the strength of F-values from ANOVA, transitioning from light red for weak effects to dark red for strong group differences. Significance levels are indicated as ∗ <0.05 and ∗∗ <0.01. TNFa = tumor necrosis factor alpha; IL6 = interleukin 6; CRP = c-reactive protein.

Obesity and oral health are closely linked and especially inflammatory factors are further supposed to drive alterations in taste perception under obese conditions [[42], [43], [44]]. Therefore, we further focused our analyses on oral health related factors, systemic inflammation and taste recognition. A significant difference between PSR scores was found for let-7a-5p (F = 5.3, P = 0.0271), miR-21-5p (F = 6.6, P = 0.0166), miR-26a-5p (F = 6.2, P = 0.0192) and miR-375-3p (F = 6.5, P = 0.014). In addition, blood levels of the inflammatory marker TNFa are related to the expression of miR-148a-3p (r = 0.4, P = 0.0326), miR-3168 (r = 0.5, P = 0.0033) and miR-320 d (r = −0.3, P = 0.048), while miR-3976 shows a strong positive correlation with blood CRP levels (r = 0.7, P = 0.0019). A negative relationship between the number of fungiform taste buds on the anterior tongue was observed for miR-619-3p (r = −0.5, P = 0.043) and miR-3960 (r = −0.5, P = 0.0437). Furthermore, several microRNAs are associated with adiponectin levels (miR-122-5p: r = −0.5, P = 0.0153; miR-619-3p: r = 0.6, P = 0.013) and insulin levels (miR-1246: r = 0.4, P = 0.0035; miR-203a-3p: r = −0.5; P = 0.018; miR-6816-5p: r = −0.3, P = 0.036) in saliva. Salivary EV-derived microRNAs exhibited strong differences among individuals with varying taste sum scores (miR-26a-5p: F = 6.6, P = 0.0159; miR-6816-5p: F = 4.9, P = 0.034). Certain microRNAs are linked to differences in the recognition of individual taste qualities, including bitter (miR-26a-5p: F = 4.9, P = 0.035; miR-6816-5p: F = 6.2, P = 0.0163), sweet (miR-3976: F = 4.8, P = 0.0381; miR-4443: F = 4.4, P = 0.0492), salty (miR-6785-5p: F = 6.7, P = 0.0188), sour (miR-6728-5p: F = 4.7, P = 0.0396), and umami (miR-3168: F = 4.3, P = 0.0424). Figure 4 (with statistics in Supplementary Table 3) provides an overview of all detected microRNAs, whereas Supplementary Fig. 2 depicts only the miRNAs emphasized in the text.

### 3.5 Link between salivary EV-derived microRNAs with eating behaviour and food intake

**Figure 5:**
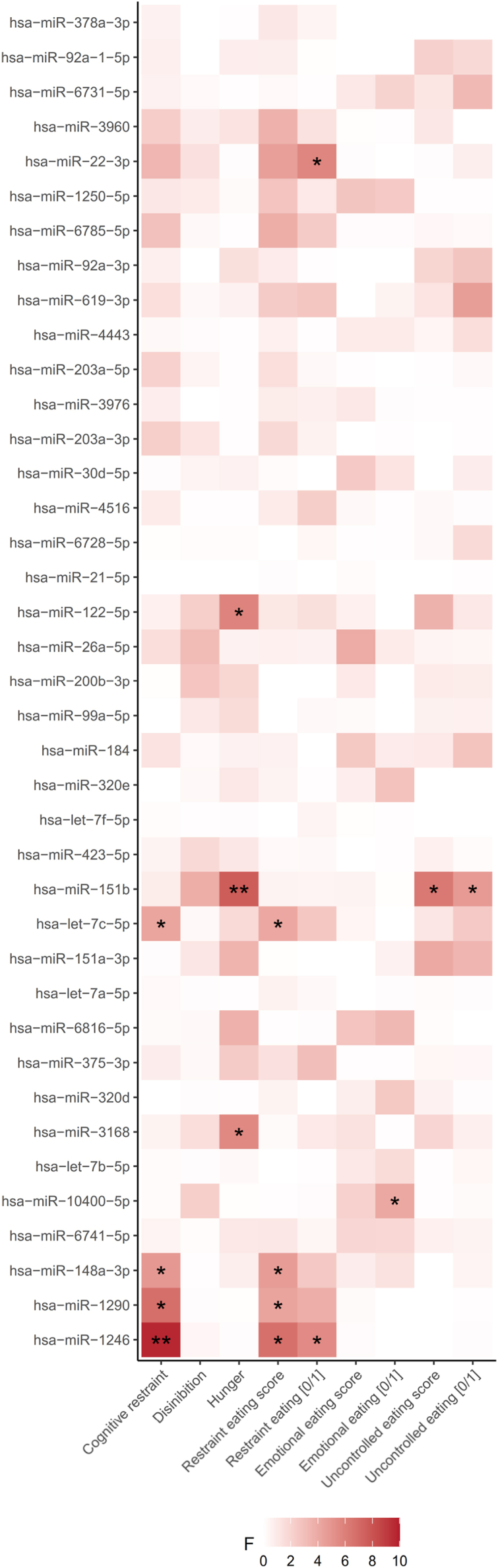
**Assessment of group differences in salivary EV-derived miRNAs for eating behavior traits.** ANOVA analyses were conducted to identify differences in salivary EV-miRNA expression between groups of different eating behavior traits or scores. Restraint-, emotional- and uncontrolled eating were further included as dichotomous variables with 0 (no) and 1 (yes). A total of 39 salivary EV-enriched microRNAs were included in the analysis. Shades of red indicating the strength of F-values from ANOVA, transitioning from light red for weak effects to dark red for strong group differences. Significance levels are indicated as ∗ <0.05 and ∗∗ <0.01.

Metabolic alterations and oral health issues during obesity have previously been linked to altered taste cell function with consequences on taste perception. As this is further influencing food consumption and eating habits, we addressed the question whether there are additional relationships between salivary EV-enriched microRNAs with eating behaviour traits and food intake. Indeed, the counts of the top three expressed microRNAs vary between groups of different cognitive restraint and/or restraint eating levels. In detail, miR-1246 is related with cognitive restraint (F = 9.7, P = 0.0031), restraint eating (F = 5.5, P = 0.0235) and the restraint eating score (F = 6.7, P = 0.0125). MiR-1290 relates with cognitive restraint (F = 6.8, P = 0.0122) and the restraint eating score (F = 4.3, P = 0.0435) while miR-148a-3p is also related with cognitive restraint (F = 4.8, P = 0.0327) and the restraint eating score (F = 4.6, P = 0.0366). Furthermore, let-7c-5p is related to cognitive restraint (F = 4.2, P = 0.0474) and the restraint eating score (F = 4.1, P = 0.0495) as well as miR-22-3p with restraint eating (F = 5.7, P = 0.0342). A relationship was found between microRNA miR-151-b and hunger (F = 7.6, P = 0.0097) and uncontrolled eating (F = 4.7, P = 0.0372; score: F = 6.3, P = 0.0174), while hunger was further related to miR-3168 (F = 5.6, P = 0.0229) and miR-122-5p (F = 5.8, P = 0.026). People with emotional eating behaviour differ in their level of miR-10400-5p (F = 4.1, P = 0.0485) from those not presenting emotional eating. All microRNAs are summarized in Figure 5 and Supplementary Table 4, while Supplementary Fig. 3 presents only those discussed in the text.

**Figure 6:**
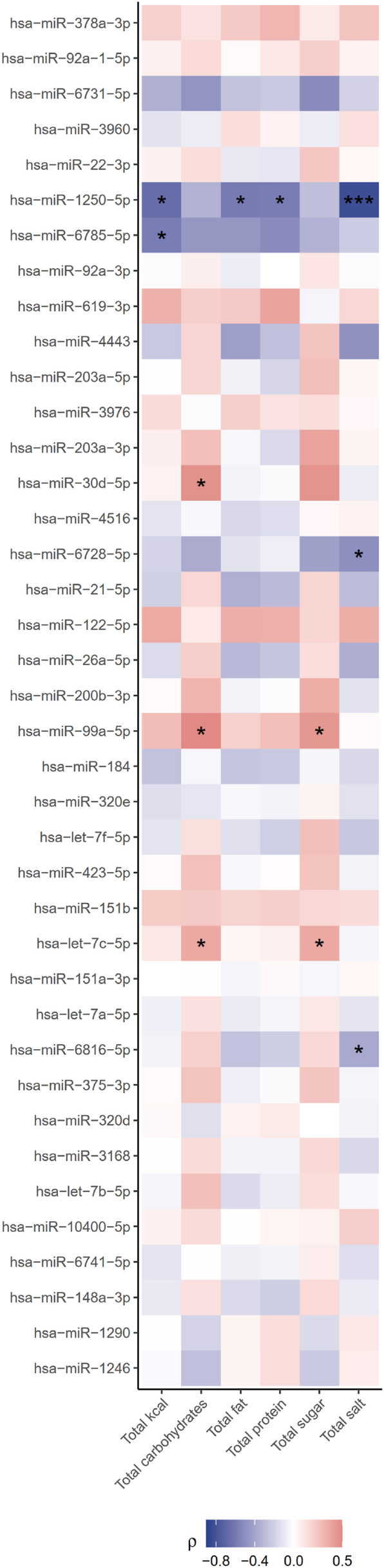
**Correlations of salivary EV-derived miRNAs with food intake.** Spearman correlation analyses were performed for 39 expressed salivary EV-derived microRNAs with selected food intake counts and total kilocalorie (kcal) intake. Strength of the correlation coefficients is color-coded, ranging from negative (blue) to positive (red). Significance levels are indicated as ∗ <0.05, ∗∗ <0.01 and ∗∗∗ <0.001, respectively.

Interestingly, we also found several correlations between salivary EV-derived microRNAs and food intake of which miR-1250-5p presents the strongest negative association with total salt intake (r = −0.8, P = 0.0009), but is also negatively correlated with total kcal (r = −0.7, P = 0.016), fat (r = −0.6, P = 0.036) and protein intake (r = −0.6, P = 0.0381). Moreover, miR-6816-5p (r = −0.4, P = 0.0245), miR-6728-5p (r = −0.5, P = 0.0266), and miR-6816-5p (r = −0.4, P = 0.0245) are correlated with lower salt intake. The two microRNAs, let-7c-5p and miR-99a-5p, exhibit positive correlations with total carbohydrate intake (let-7c-5p: r = 0.4, P = 0.038; miR-99a-5p: r = 0.5, P = 0.015) as well as sugar intake (let-7c-5p: r = 0.4, P = 0.0377; miR-99a-5p: r = 0.4, P = 0.0377). Additionally, the microRNA miR-30 d-5p shows a positive association with total carbohydrate intake (r = 0.5, P = 0.0475), while miR-6785-5p shows a negative correlation with total caloric intake (r = −0.6, P = 0.0489). An overview of all detected miRNAs is shown in Figure 6, with the corresponding statistics provided in Supplementary Table 5. Supplementary Fig. 4 displays only the miRNAs highlighted in the main text.

## 4 Discussion

Salivary EVs hold promising potential for their use as biomarkers also in systemic diseases while offering advantages over blood and tissue samples by their non-invasive collection. Previously we linked the number of salivary EVs to obesity and body fat distribution [7]. In the present study, we examined EV's microRNA content to explore connections with taste perception, eating behaviour, and metabolic and oral health.

Similar to other studies, small RNA sequencing revealed that a significant proportion of the reads were either too short or could not be mapped to the human reference genome, with certain regions highly represented across all samples [37,40,44]. Additionally, approximately 57 % of the mapped reads could not be classified into specific RNA types. This is likely due to a larger proportion of uncharacterized microRNAs and other RNA types, as well as contamination from bacterial, viral, and archaeal RNA, which are typically present in human saliva [45,46]. To address these challenges and mitigate the pitfalls associated with small RNA sequencing, more sophisticated annotation methods need to be developed or implemented, which is beyond the scope of this study [35,46]. In the remaining dataset, we observed a high abundance of rRNA which has been shown to depend highly on the cell type origin of EVs [47]. Furthermore, only a small proportion of microRNAs and other small, non-coding RNAs were found beside approximately seven % of mRNAs, indicating that a high proportion of larger vesicles, have been analyzed, as it has been shown that especially microvesicular bodies contain more rRNA with a lesser degree of small RNAs, while exosomes, are thought to be loaded with microRNAs in a selective and directed manner [47,48]. We did not separate between small and large EVs due to restricted sample material and the aim to address an overall broad spectrum of salivary EVs, in order to promote their overall potential as biomarkers. However, one needs to keep in mind that the potential functional properties of small and large EVs are very distinct due to differences in their biogenesis and content [49]. In addition, others have shown a higher degree of especially microRNAs in EVs derived from saliva, which might partially be explained by the large discrepancies in EV preparations and following analyses steps [48,50]. In general, there are huge differences between studies which might also arise from the cell type/source being studied, hence cellular origin of EVs, variations in EV and RNA isolation methods applied up to diverse sequencing platforms and protocols used for library preparation. By studying saliva, an additional circumstance might arise from the collection of unstimulated or stimulated saliva. As these variations in the study of EVs are a common problem making it hard to compare results and draw conclusions, we strictly followed the “minimal information for the study of extracellular vesicles” (MISEV) criteria, which is of high relevance and allows more comprehensive and useful data evaluation [51]. In any case, studying the cargo of salivary EVs is of high interest for our understanding of their contribution to oral health and taste function in relation to metabolism. Therefore, we further focused on the microRNA proportion found in the salivary EVs which comprise 626 microRNAs. Pathway enrichment analyses revealed that 82 % are related to endocytosis, a fundamental process for cellular uptake including receptor-ligand uptake in general, which is also necessary for food substances to be taken up by taste receptors [15,41]. Endocytosis is initiated in a ligand-specific manner which holds true for taste receptors such as the sweet modulating TAS1R2/TAS1R3 complex, hence affecting its desensitization uppon ligand binding [41]. It has previously been shown that microRNAs affect this process with subsequent consequences on signaling pathways but also on stem cell lineage commitment, all processes being involved in taste bud signaling and homeostasis [52]. Specific miRNAs, like miR-200a/b/c, were associated with endocytosis and FGF signaling, suggesting roles in taste bud development and function [15]. Among the top enriched pathways are leptin and insulin signaling, adipogenesis, but also various cellular signalling mechanisms, such as mTOR-, MAPK-, WNT- or PPAR-signaling, and inflammatory processes indicating their involvement in metabolic regulation. Adipose tissue is a major source of circulating EVs rich in microRNAs that influence metabolic pathways and eating behavior, contributing to obesity-related dysfunctions. These EVs may reach the oral cavity via circulation, while obesity-induced local changes, such as altered salivary flow and composition, may further impact salivary EV concentration and content.

Our analysis identified 87 microRNAs enriched in the leptin signaling pathway. Leptin, produced by salivary glands, influences taste perception and is affected by obesity [[53], [54], [55]]. It dampens sweet signals by reducing ATP-channel activity in taste cells. Knockdown of leptin in these cells decreases fatty acid detection and also affects the proliferation of non-taste oral keratinocytes [56,57]. Thus, microRNA-mediated regulation of oral leptin may play a key role in both taste signaling and oral health. MiR-203a-3p, which is predicted to directly target leptin, is enriched in pathways related to leptin and insulin signaling, type 2 diabetes, and taste perception, among others in our data. However, no correlations with taste were observed for miR-203a-3p in the current dataset, but a negative significant correlation with BMI, C-peptide levels and salivary insulin levels. In addition to leptin, insulin influences taste cell proliferation and enhances salty taste by modulating ENaC (epithelial sodium channel) and Na/K/ATPase activity [58,59]. In rodents, miR-203a-3p levels change sharply after weaning — a key stage in β-cell maturation and insulin production [60]. Persistent expression of this microRNA can limit β-cell proliferation into adulthood. It remains to be elucidated whether salivary EV-derived miR-203a-3p affects local adipokine production, impacting cell proliferation and taste perception.

Interestingly, we found a relationship of miR-6816-5p, with salivary insulin levels, which is further related to salt intake, while only a trend towards a correlation with salty taste score was shown. However, it is explaining differences in the bitter and total taste scores. Future studies may focus on this microRNA and its contribution to taste modulation, food consumption and how local factors such as salivary insulin might contribute in this context. The strongest association with dietary intake was shown for miR-1250-5p, while miR-26a-5p is of particular interest reagarding its relation with taste ability. Participants with different taste sum and bitter scores have distinct miR-26a-5p level and this microRNA is further related to BMI, blood glucose level and the PSR. MiR-26a-5p has been extensively studied, primarily for its oncogenic potential while it seems to be implicated in osteoarthritis and diabetic nephropathy, suggesting an influence on various pathways [61]. In general, little is known about the role of microRNAs in the fundamental mechanisms of taste perception within oral taste tissues, highlighting a potential area for future research. However, that dietary intake can alter microRNA composition has been well known [[62], [63], [64], [65]]. While we did not find associations with measures [22,26] of food preference, food craving, food addiction or current hunger (data not shown) we could demonstrate a strong potential of the top three enriched microRNAs to explain a variation in some trait eating behaviours, namely cognitive restraint and restraint eating. Although not associated with taste recognition in the current cohort, those microRNAs are strongly associated with anthropometrics and also fasting glucose, HOMA-IR, the level of high density lipoprotein (HDL) cholesterol, free fatty acids as well as blood pressure and are enriched in several of the identfied pathways. The miR-1246 is further related to increased leptin serum levels and elevated salivary insulin levels. MiR-2146 and miR-148a-3p originating from adipose tissue EVs regulate glucose homeostasis, lipid metabolism and inflammation in distant tissues [2]. Moreover, miR-1246 from adipocyte-derived EVs promotes macrophage polarization towards a pro-inflammatory state, exacerbating insulin resistance. Silencing miR-1246 reverses cell cycle arrest in metabolic stress models, while restoring miR-148a-3p improves insulin secretion in diabetic β-cells [66,67].

Overall, the highly expressed 39 microRNAs are related to either metabolic factors and eating behaviour or taste recognition separately, suggesting diverse routes of action and contribution to those phenotypes, by being present at high level in salivary EVs additionally to blood. Concrete effects on tissues in the oral cavity have scarcely been addressed so far. Salivary miR-1246 and miR-1290 are promising non-invasive biomarkers for oral and metabolic diseases [68]. MiR-1290, linked to head and neck cancer, was previously found in salivary EVs of cancer patients [69]. In our non-cancer cohort, its presence suggests limitations in using it solely as a cancer marker. However, since obesity is a risk factor for cancer, elevated levels of these microRNAs may have prognostic value. Some may reflect obesity-related metabolic changes and serve as biomarkers, while others might influence local tissues like taste cells without systemic effects. However, microRNAs in general are well known to contribute to eating behaviour by influncing brain circuits of energy regulation, food consumption and areas involved in the formation of an appetitive motivation to eat [[70], [71], [72]]. Obesity is linked to poor oral health and increased periodontal diseases which is concomitant with changes in the bacterial composition of the oral niches leading to inflammatory processes [11,73]. Among the microRNAs being altered in saliva of periodontits patients is miR-148a-3p, which was associated with increased serum TNFa level in the present cohort [74,75]. Although miR-148a-3p does not explain variations in the PSR score in this study, other microRNAs are related including miR-26a-5p. The discrepancys might partially be explained by the current study participants scoring low on the PSR code, meaning they are not defined as patients with increased risk for periodontitis. However, an enrichment of salivary EV-derived microRNAs being related to inflammation, as well as systemic metabolic health and eating behaviour might point towards their strong regulatory role in all of these processes and may contribute to explain how obesity interconnects these pathways. To address whether salivary derived EVs internalize and affect local cells of the tongue, we treated human Fungiform Taste Cells (hFTC) with isolated EVs from lean people (see Supplementary Material). Results indicate that salivary EVs are, at least partially, taken up by oral cells (Supplementary Fig. 5) and are of relevance for transcriptional changes of genes potentially involved in taste signaling, the inflammatory response and cell-stability or differentiation (Supplementary Fig. 6) [20,[76], [77], [78], [79]]. However, we observed a great inter-individual variance of mRNA expression after EV-treatment which currently limits interpretation and generalization of the findings. Additionally, these experiments do not allow us to conclude that the observed gene expression changes are driven by microRNAs contained in EVs. Nevertheless, they clearly demonstrate that salivary EVs exert functional effects on tongue-derived cells. Future studies focusing on individual microRNA-mRNA target interactions, applying functional target-site cloning strategies or targeted microRNA loading of EVs, are needed to address specific functions of the identified and enriched salivary EV-derived microRNAs. Further, it needs to be addressed whether salivary EV-derived microRNAs might reach the brain via systemic circulation and effect eating behaviour without affecting taste sensation.

## 5 Conclusion

The current analyses addressed the microRNA composition of salivary EVs in a cohort of normal weight to participants with overweight and obesity. Overall, the identified microRNAs are enriched in pathways related to metabolism, adipocytokine function, taste and cellular signaling as well as inflammation. Top enriched microRNAs are related to anthropometrics and metabolic parameters and explain variations in trait eating behaviour, taste recognition and food intake. These interconection of EVs, obesity and taste related traits have been evaluated in one of the largest cohorts availble for the study of salivary EVs and provides important new connections of EV derived microRNAs with the aforementioned paramters. This will help establishing better prevention and treatment strategies for this burgening health concern.

## 6 Strength and limitation

Our study is limited in several aspects. A very low amount of saliva as starting material for EV isolation, did not allow us to discriminate between small and large EVs or to use pull-down analyses for a very strict identification of EVs. Rather all particles in the solution have been counted as EVs. However, the efficient isolation of EVs has been strictly evaluated earlier with a variety of available methods and highlights the possibility of using small saliva amounts for diagnostics [7]. The use of the overall spectrum of salivary EVs for disease/phenotype characterization analyses is beneficial with regard of their overall diagnostic potential. Another limitation arises from the variability in RNA yield and sequencing depth across saliva-derived EV libraries, which is a known challenge in small RNA-seq studies [36]. To address this, we applied stringent filtering and TMM normalization to minimize technical bias. While this strategy ensures robust expression estimates, it may also have excluded low-abundance miRNAs that could be biologically relevant. Furthermore, the number of participants included in this study is rather small for population-based research. However, despite the outlined limitations, we present, to the best of our knowledge, data from one of the largest cohorts available in which salivary EVs have been characterized and related to metabolic alterations occurring in obesity. This may also be reflected by the high number of microRNAs found in the salivary EVs in our analyses compared to others.

## CRediT authorship contribution statement

**Kristin Röhrborn:** Writing – review & editing, Writing – original draft, Methodology, Investigation, Formal analysis. **Anne Hoffmann:** Writing – review & editing, Writing – original draft, Visualization, Software, Methodology, Investigation, Data curation. **Andrea Lorenz:** Writing – review & editing, Methodology, Investigation. **Peter Kovacs:** Writing – review & editing, Supervision. **Tobias Hagemann:** Writing – review & editing, Formal analysis, Data curation. **Paul Czechowski:** Writing – review & editing, Formal analysis. **Maria Sehm:** Writing – review & editing, Investigation. **Annette Horstmann:** Writing – review & editing, Investigation. **Michael Stumvoll:** Writing – review & editing, Resources, Project administration. **Matthias Blüher:** Writing – review & editing, Resources, Project administration. **Imke Schamarek:** Writing – review & editing, Project administration, Conceptualization. **Kerstin Rohde-Zimmermann:** Writing – review & editing, Writing – original draft, Supervision, Project administration, Funding acquisition, Conceptualization.

## Contributors

KRZ and IS designed the study, enrolled participants and did data acquisition of the OTB-cohort. KR performed laboratory experiments and did data analyses. KRZ and KR wrote the draft of the manuscript. AH performed raw data processing and performed data analyses. AL is responsible for dental screening. TH and PC contributed to raw data processing. PK, MB and MS supported the study setup. MS and AHorstmann enrolled participants of the MPI-cohort. All authors contributed to discussions and finalizing the manuscript draft.

## Data sharing statement

All data from this study are contained within the published article and its supplementary information files. The small RNA sequencing data can be accessed at NCBI's Sequence Read Archive [DOI: 10.1093/nar/gkq1019] under BioProject ID PRJNA1257279 (https://www.ncbi.nlm.nih.gov/bioproject/PRJNA1257279).

## Funding

Project grant from the Else Kröner-Fresenius Foundation to KRZ (2021_EKEA.30). Project grants from the Medical Faculty of the University of Leipzig to KRZ (IFBADI-042) and IS.

## Declaration of competing interest

The authors declare to have no conflict of interest except MB who received honoraria as a consultant and speaker from Amgen, AstraZeneca, Bayer, Boehringer-Ingelheim, Daiich-Sankyo, Lilly, Novo Nordisk, Novartis, Pfizer, and Sanofi.

## Data Availability

Data will be made available on request.
