# Supplementary material for: Salivary extracellular vesicle-derived microRNAs are related to variances in parameters of obesity, taste and eating behaviour
Source: Mol Metab. 2025 Oct 3;102:102265. doi: 10.1016/j.molmet.2025.102265 (PMC12557581; doi:10.1016/j.molmet.2025.102265)
Supplement: Multimedia component 2 [file mmc2.docx]

**Supplementary Figure 1: Detailed associations of salivary EV-miRNAs with metabolic traits.** Associations (P < 0.05) of selected salivary EV-derived microRNAs with metabolic traits, obesity status, and gender. Box plots from ANOVA depict differences across obesity status and gender, while regression plots based on Spearman’s correlation analyses illustrate associations with metabolic traits. HOMA-IR = Homeostasis Model Assessment-Insulin Resistance; BMI = body mass index.

**Supplementary Figure 2: Detailed associations of salivary EV-miRNAs with oral health, systemic inflammation and taste recognition.** Associations (P < 0.05) of selected salivary EV-derived microRNAs with periodontal screening and recording (PSR) score, systemic inflammation and taste recognition. Box plots from ANOVA depict differences across PSR score and taste recognition, while regression plots based on Spearman’s correlation analyses illustrate associations with systemic inflammation. TNFa = tumor necrosis factor alpha; CRP = c-reactive protein.

**Supplementary Figure 3: Detailed associations of salivary EV-miRNAs with eating behaviour traits.** Box plots from ANOVA (P < 0.05) of selected salivary EV-derived microRNAs with eating behaviour traits.

**Supplementary Figure 4: Detailed correlations of salivary EV-miRNAs with food intake parameters.** Regression plots based on Spearman’s correlation analyses (P < 0.05) of selected salivary EV-derived microRNAs with food intake and total kilocalorie (kcal) intake are illustrated.


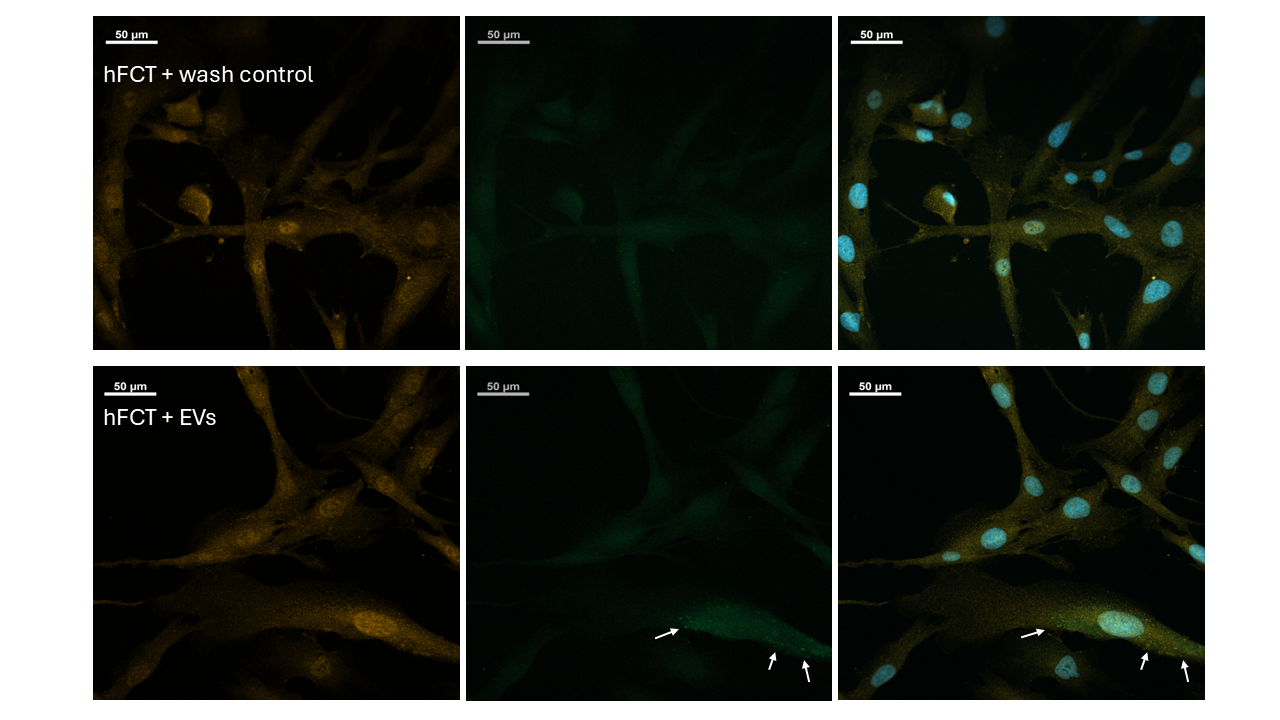


**Supplementary Figure 5:** **EV-uptake into human fungiform taste cells.** To delve into the functional relevance of salivary EVs on cells of the oral cavity, we proofed if isolated EVs are taken up by a commercially available human fungiform taste cell line (hFTC). After treating the cells for 15 min, EVs were partially taken up by the cells. Representative confocal laser scanning images are shown (x-y top view). Upper panel: hFCT loaded with washing controls without EVs (PKH67 dye in PBS) to verify excess dye removal using ultrafiltration. Lower panel: PKH67-labeled EVs taken up from hFTC. White arrows point towards internalized EVs. Cells were labeled with KRT8 (orange), EVs were labeled with PKH67 membrane dye (green) and cell nuclei were stained with DAPI (blue). Scale bars represent 50µm. EVs = extracellular vesicles.

*
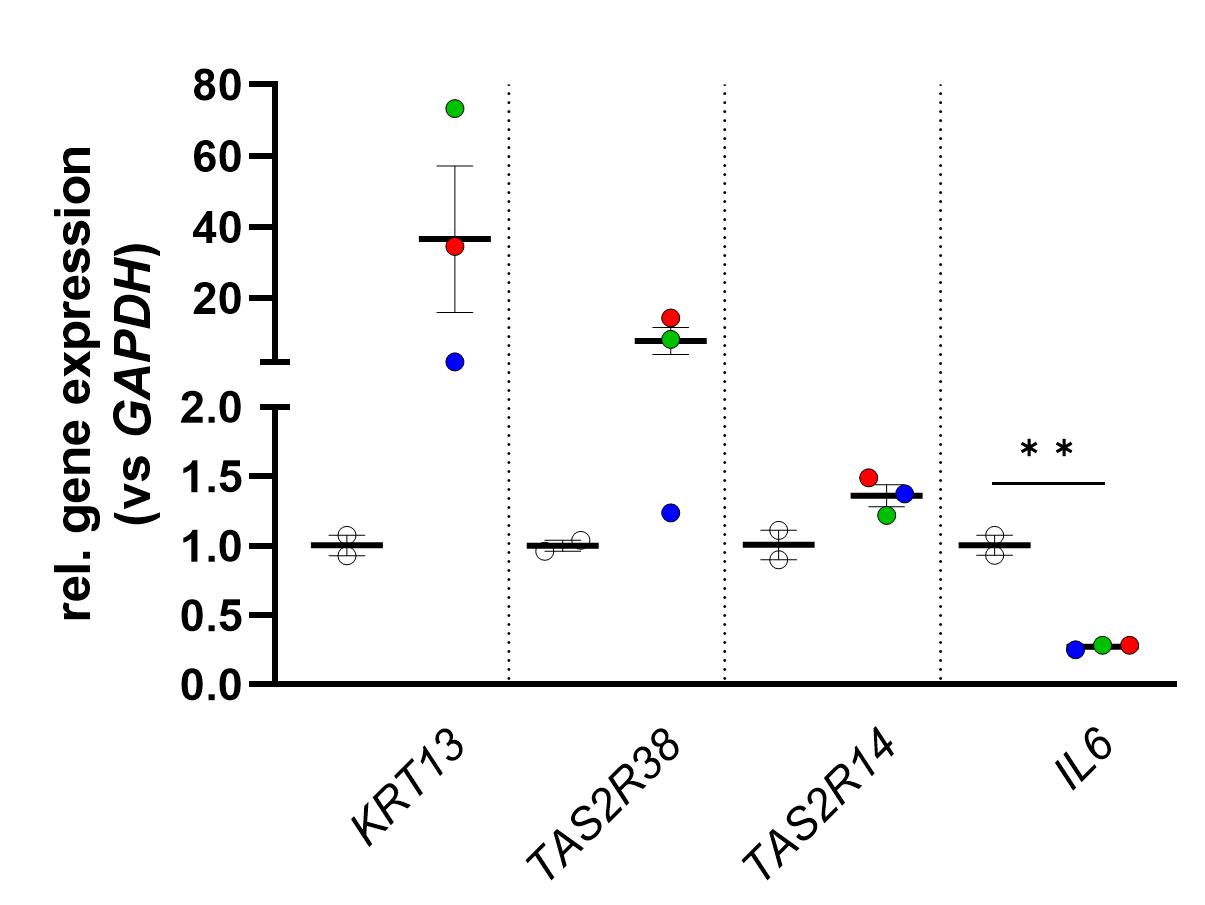
*

**Supplementary Figure 6:** **Alterations of gene expression in hFTC after EV-treatment.** As our results imply a role of EV-microRNAs in taste recognition but also inflammatory processes among others, we checked for alterations in the levels of TAS2R38, TAS2R14 and IL6, previously shown to play a role in those mechanisms in gustatory and non-gustatory cells ^20,76_79^. hFTC were treated with 3.45x10^8^ EV-particles (per sample) isolated from three lean individuals marked in green, red and blue. Two non-treatment controls (cells incubated with PBS) were included and marked with white circles. Gene expression is presented relative to the non-treatment controls (ΔΔCT) and normalized to GAPDH. Differences in gene expression between the mean of EV-treated cells and non-treatment controls was calculated using student’s t-test. P-values < 0.05 were considered significant and highlighted as ** P < 0.01. hFTC = human Fungiform Taste Cells; *KRT13* = cytokeratin 13; *TAS2R38* = Taste 2 Receptor Member 38; *TAS2R14* = Taste 2 Receptor Member 14; *IL6* = Interleukin 6; *GAPDH* = Glyceraldehyde-3-Phosphate Dehydrogenase

**Supplementary methods - Cell culture analyses**

To address functional consequences of salivary derived EVs on gene transcription, we used saliva samples from a comparable cohort recruited in collaboration with the Max-Planck-Institute for Human Cognitive and Brain Sciences, Leipzig, Germany which is described in detail elsewhere ^38^. Saliva collection and EV isolation followed the same procedures as described for the OTB-participants. The immortalized human fungiform taste cell line (hFTC; abm, Richmond, BC. Canada) was taken as cell model and cells were cultured in PriGrow V Medium (abm, Richmond, BC. Canada) supplemented with 10 % fetal bovine serum and 1% penicillin/streptomycin under standard conditions. Before conducting experiments, PBS and culture media were depleted of exosomes by using ultracentrifugation at 100.000 x g and 4°C overnight. For proofing EV internalization, cells were cultured until 60 % confluence on 8-well chamber slides (Ibidi, Gräfeling, Germany). Isolated EVs from one participant were incubated with 1 µl PKH67-dye (PKH67 Green Fluorescent Cell Linker Mini Kit) for membrane labelling as per manufacturers recommendations (Sigma Aldrich, Darmstadt, Germany). Excess dye was removed by transferring the sample to Vivaspin-500 300 kDa MWCO ultrafiltration columns (Sartorius, Göttingen, Germany) and washing samples three times with 500 µl PBS each. PBS-washing controls were included to evaluate excess dye removal. Cells cultured on the 8-well chamber slides were incubated with washing control or labelled EVs in 300 µl culture media per well. After 15 min, cells were washed twice with PBS and fixed with 4% paraformaldehyde for 30 min. To visualize the cells, guinea pig anti-KRT8 (1:250; BP5007, Origene Herford, Germany) and anti-guinea pig Cy3 (1:100; AP108C, Sigma Aldrich, Darmstadt, Germany) staining was applied following permeabilization (0.5 % Triton X-100 in PBS) and blocking (10 % FBS and 0.1 % Triton X-100 in PBS) the cells with standard procedures. Cell nuclei were stained by applying mounting medium containing DAPI (Ibidi, Gräfeling, Germany) to each well. EV uptake was evaluated using a LSM 980 KMAT confocal laser scanning microscope (ZEISS, Oberkochen, Germany) and images were processed using ZEN 3.5 blue edition software (ZEISS, Oberkochen, Germany). The setting was kept constant for image generation and processing. To evaluate effects of EV-treatment on gene expression, EV-samples from three individual participants matched for BMI (21.7 ± 0.9), age (24 ± 1) and gender (men) were prepared. Cells were cultured on 6-Well plates until 60 % confluence and either non-treated (loaded with 1 ml PBS + 2 ml media) or incubated for 24 h with 3.45 x 10^8^ EV-particles per well. RNA extraction was performed with Trizol/Chloroform and cDNA was generated from 500 ng RNA per sample using SuperScript III RT (Invitrogen, Darmstadt, Germany) applying manufacturers recommendations. Inclusion of non-template and empty reverse transcriptase controls served as quality control. Finally, quantitative real-time PCR (RT-qPCR) was performed with the PowerUp SYBR Green Master Mix (Thermo Fisher, Darmstadt, Germany) and samples were run on a QuantStudio 6 Real Time PCR System (Thermo Fisher, Darmstadt, Germany) in triplicates. Gene expression was calculated using the ΔΔCT method and normalized to the level of *GAPDH*. Differences in gene expression between the mean of EV-treated cells and non-treatment controls was calculated using student’s t-test and P-values < 0.05 were considered significant.
